# The infective causes of hepatitis and jaundice amongst hospitalised patients in Vientiane, Laos

**DOI:** 10.1016/j.trstmh.2010.03.002

**Published:** 2010-07

**Authors:** Bounkong Syhavong, Bouachanh Rasachack, Lee Smythe, Jean-Marc Rolain, Anne-Marie Roque-Afonso, Kemajittra Jenjaroen, Vimone Soukkhaserm, Simmaly Phongmany, Rattanaphone Phetsouvanh, Sune Soukkhaserm, Te Thammavong, Mayfong Mayxay, Stuart D. Blacksell, Eleanor Barnes, Philippe Parola, Elisabeth Dussaix, Didier Raoult, Isla Humphreys, Paul Klenerman, Nicholas J. White, Paul N. Newton

**Affiliations:** a Wellcome Trust-Mahosot Hospital-Oxford Tropical Medicine Research Collaboration, Microbiology Laboratory, Mahosot Hospital, Vientiane, Lao PDR; b WHO/AO/OIE Collaborating Centre for Reference & Research on Leptospirosis, Queensland Health Forensic and Scientific Services, Coopers Plains, Queensland, 4108, Australia; c URMITE CNRS-IRD UMR 6236, Faculté de Médecine et de Pharmacie, Université de la Méditerranée, Marseille, France; d AP-HP, National Reference Centre for HAV, Hôpital Paul Brousse, Villejuif, 94804 France; e Faculty of Tropical Medicine, Mahidol University, 420/6 Rajvithi Rd., Bangkok 10400, Thailand; f Centre for Clinical Vaccinology and Tropical Medicine, University of Oxford, Churchill Hospital, Oxford, UK; g National Blood Transfusion Centre, Lao Red Cross, Vientiane, Lao PDR; h Faculty of Post Graduate Studies, University of Health Sciences, Vientiane, Lao PDR; i The Peter Medawar Building for Pathogen Research, University of Oxford, Oxford, UK

**Keywords:** Jaundice, Hepatitis E virus, *Orientia tsutsugamushi*, *Rickettsia typhi*, Leptospirosis, Laos

## Abstract

There is little information on the diverse infectious causes of jaundice and hepatitis in the Asiatic tropics. Serology (hepatitis A, B, C and E, leptospirosis, dengue, rickettsia), antigen tests (dengue), PCR assays (hepatitis A, C and E) and blood cultures (septicaemia) were performed on samples from 392 patients admitted with jaundice or raised transaminases (≥ × 3) to Mahosot Hospital, Vientiane, Laos over 3 years. Conservative definitions suggested diagnoses of dengue (8.4%), rickettsioses (7.3%), leptospirosis (6.8%), hepatitis B (4.9%), hepatitis C (4.9%), community-acquired septicaemia (3.3%) and hepatitis E (1.6%). Although anti-hepatitis A virus (HAV) IgM antibody results suggested that 35.8% of patients had acute HAV infections, anti-HAV IgG antibody avidity and HAV PCR suggested that 82% had polyclonal activation and not acute HAV infections. Scrub typhus, murine typhus or leptospirosis were present in 12.8% of patients and were associated with meningism and relatively low AST and ALT elevation. These patients would be expected to respond to empirical doxycycline therapy which, in the absence of virological diagnosis and treatment, may be an appropriate cost-effective intervention in Lao patients with jaundice/hepatitis.

## 1 Introduction

Patients with biochemical liver impairment and an infective aetiology are commonly admitted to hospital in the tropics with a multitude of causes that may be difficult to distinguish. In Southeast Asia the aetiology includes pathogens such as hepatitis A, B, C and E, dengue, leptospirosis, typhus and those causing septicaemia.^1^, ^2^ Recent work in Vientiane, Lao PDR (Laos), has emphasised the importance of *Salmonella enterica* serovar typhi, scrub typhus (*Orientia tsutsugamushi*) and murine typhus (*Rickettsia typhi*) as causes of fever.^3^, ^4^ A study of the aetiology of jaundice and dark urine among 280 patients admitted to Vientiane hospitals gave evidence of acute hepatitis A virus (HAV) and B virus (HBV) infection in 14% and 10% of cases, respectively.^1^ Among Vientiane blood donors the prevalences of hepatitis B surface antigen (HBsAg) and anti-hepatitis C virus (HCV) antibody were 8.7% and 1.1%, respectively.^5^

Whilst investigating the causes of fever among hospitalized patients in Vientiane a substantial proportion were jaundiced or had raised transaminases. Most studies of the causes of jaundice or hepatitis have concentrated on a narrow range of pathogens, for example the hepatitis viruses, in one population and not cast the diagnostic net broad to include viruses, rickettsia, leptospires and conventionally culturable bacteria. We therefore investigated the aetiology amongst inpatients to try to capture the diversity of organisms responsible. As serological tests may not be sufficiently specific in populations with multiple potential etiologies because of the persistence of antibody, we endeavored to determine the aetiology using specific tests based on rises in antibody titre between acute and convalescent samples and antigen and PCR assays.

## 2 Patients and methods

### 2.1 Study site and patients

The study was conducted at Mahosot Hospital, Vientiane, a 400-bed primary-tertiary hospital with approximately 1200 admissions/month. This, along with four other major hospitals of 1210 beds, and local hospitals, serves a population of approximately 900 000 people, including the urban population of Vientiane City and surrounding farming communities of Vientiane Province, and, less frequently, outlying provinces.

Patients of any age admitted to Mahosot Hospital with acute jaundice or elevated AST or ALT (by ≥3 times the upper limit of the reference range) who gave informed verbal consent were recruited (May 2001–May 2004) on eight adult and paediatric wards (192 beds). Blood samples were taken for admission and convalescent sera and, if community-acquired septicaemia was suspected, for a pair of blood cultures.^3^ If the patient came from an area of Laos with endemic malaria, Giemsa-stained malaria thick and thin films were examined.

### 2.2 Laboratory procedures

Blood cultures were processed^3^ and full blood counts (*n* = 252) and serum biochemistry (*n* = 375) determined on Abx MICROS*OT* (Abx Hematologie) and Cobas Integra (Roche, Basel, Switzerland) analyzers, respectively. In order to provide a clinical service, all sera were tested with Vidas immunoassay tests for anti-HAV IgM immunoglobulin (bioMérieux, Marcy l’Etoile, France), HBsAg, anti-HBsAg (Serodia-HBs, Serodia-Anti-HBs; Fujirebio, Japan) and HCV antibody tests (Serodia-HCV; Fujirebio, Japan). Serum samples were stored at −80 °C. Sera from a subset of 51 patients with anti-HAV IgM were analysed by anti-HAV IgG avidity assays and HAV PCR.^6^, ^7^ The AFRIMS ELISA was used to detect IgM and total antibody (Ig) to HEV.^8^, ^9^ Acute HEV infection was defined as anti-HEV IgM≥100 Walter Reed (WR) U/ml or anti-HEV Ig ≥500 WR U/ml. Sera from all patients with definite or probable acute HEV were tested by HEV PCR.^10^ Patients positive for HBsAg or anti-HBsAg were tested using the Murex Anti-HB IgM ELISA kit (Abbott Laboratories, Abbott Park, IO, USA) for IgM anti-core antigen (HBcAb). Samples from patients with HCV antibodies were tested by HCV RNA 5′UTR PCR using the Superscript II RT (Invitrogen) and viruses genotyped.^11^ Micro-immunofluorescence (IFA) assays for *Orientia*, *Rickettsia*, *Coxiella* species and *Neorickettsia sennetsu* (Table 1) were considered positive if (1) positive antibody titers were > 1/128 for IgG and > 1/64 for IgM, or (2) seroconversion, or (3) four-fold or greater increase in titers between the acute and the convalescent serum.^4^, ^12^ In microscopic agglutination tests (MAT) for *Leptospira* antibodies, a patient was considered to have a current or recent *Leptospira* infection if serum showed a titer of ≥1:400, or if paired sera demonstrated a four-fold rise (Table 1).^13^ Serum anti-dengue IgM/IgG antibodies and dengue NS1 antigen were assayed using ELISA kits (IgM/IgG Duo, Early Dengue, PanBio, Australia) for 189 patients without a confirmed diagnosis.

**Table 1:** Serology, antigen and gene detection markers of acute jaundice/hepatitis among 392 Lao patients at Mahosot Hospital 2001–2004

Marker	All patients	Fever
	No. positive/tested (%)	No. positive/tested (%)
Hepatitis A		
IgM	136/380 (35.8)	115/300 (38.3)
IgM w/o other diagnosis	110/380 (28.9)	95/300 (31.7)

Hepatitis B		
HBsAg	70/389 (18.0)	50/304 (16.5)
Anti-HBsAg	61/388 (15.7)	51/303 (16.8)
Anti-IgM core	19/119 (16.0)	11/93 (11.8)

Hepatitis C		
Total Ig	63/389 (16.2)	46/304 (15.1)
PCR	19/50 (38.0)	13/36 (36.1)

Hepatitis E		
IgM	6/378 (1.6)	5/298 (1.7)
PCR	2/12 (17.0)	2/11 (18.2)

Dengue		
IgM, IgG and NS1	33/189 (17.5)	30/139 (21.6)
Primary	14/189 (7.4)	13/139 (9.4)
Secondary	19/189 (10.0)	17/139 (12.2)

Leptospirosis^a^		
MAT	26/385 (6.8)	24/303 (7.9)

Murine typhus^b^		
IgM/IgG IFA	14/382 (3.7)	11/301 (3.7)

Scrub typhus^c^		
IgM/IgG IFA	8/382 (2.1)	8/301 (2.7)

Spotted fever group^d^		
IgM/IgG IFA	6/382 (1.6)	5/301 (1.7)

Septicaemia		
Culture positive	13/166 (7.8)	12/146 (8.2)

Ig: immunoglobulin.

a The serovars tested were Pomona, Hardjo, Tarassovi, Grippotyphosa, Celledoni, Copenhageni, Australis, Pyrogenes, Canicola, Hebdomadis, Mini, Sarmin, Autumnalis, Cynopteri, Ballum, Bataviae, Djasiman, Javanica, Panama, Shermani and Mwalok. The serovars used in an MAT panel may not always represent the actual infecting serovar for the region but demonstrate reaction within the Serogroup housing the actual serovar.

b Using whole-cell antigens of *R. typhi*.

c Using whole-cell antigens of *O. tsutsugamushi* serotypes Karp, Kato, Gilliam, and Kawasaki.

d Using whole-cell antigens *R. conorii* subsp. indica, *R. felis, R. heilongjangensis, R. helvetica, R. honei, R. japonica, Rickettsia* ‘ATI’, *R. slovaca*.^4^

### 2.3 Statistical analysis

Analysis was performed using STATA v.10 (Stata Corp., College Station, TX, USA). Categorical variables were compared with Fisher's exact test and continuous variables by Student's *t*-test and Mann-Whitney *U* test as appropriate. Multiple logistic regression was used to identify predictor variable effects. A *P* value <0.02 was regarded as statistically significant.

## 3 Results

### 3.1 Patients

During the three years of the study 403 patients were recruited; 11 were included in error (without jaundice or transaminases three times the reference upper range). Patients came predominantly from Vientiane City (69.4%) and Vientiane Province (16.3%). The most frequent occupations (*n* = 391) recorded were housewife (16.9%), unemployed (16.1%), student (15.9%), government official (15.6%), building worker (14.3%) and rice farmer (8.7%). Three hundred and seven (78.3%) patients had a history of fever in the previous week or fever (≥37.5 °C) recorded on admission. Jaundice, or a history of jaundice in the presenting illness, were present in 334/390 (85.6%) and 199/385 (52%) had AST and/or ALT three times or above the upper limit of the local reference range (<37 IU/l and < 40 IU/l, respectively). Jaundice and transaminases raised at least threefold were both present in 174/385 (45.2%) of patients. A convalescent serum was taken from 204 (52%) patients a median (range) of 6 (1–43) days after the admission sample.

The serological, antigen-detection based and PCR-based assays of samples from 392 patients suggested, conservatively, the following diagnoses (Table 1): hepatitis B 19/389 (4.9%, IgM HBcAb positive among those HBsAg or anti-HBsAg positive), hepatitis C 19/389 (4.9%. HCV PCR and anti-HCV positive), dengue 33/189 (17.5% or 8.4% of all 392 patients) (NS1 antigen positive and/or anti-dengue IgM/IgG positive), rickettsioses 28/382 (7.3%) (IgM/IgG positive), leptospirosis 26/385 (6.8%) (MAT positive), hepatitis E 6/378 (1.6%. HEV IgM or Ig positive and/or PCR positive), and 13/166 (7.8% or 3.3% of all 392 patients) with community-acquired septicaemia. Dengue, rickettsial infections, leptopirosis and HAV tended to be more common in the monsoon and post-monsoon months whilst septicaemia, HBV and HCV occurred throughout the year.

**Figure 1:**
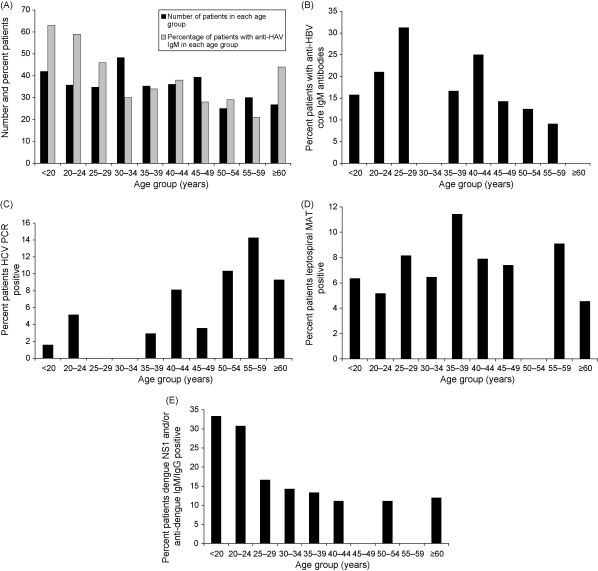
(A) Number of patients recruited per age category and percent age distribution of patients with anti-HAV IgM antibodies; (B) percent age distribution of patients with anti-HBV core IgM antibodies; (C) percent age distribution of patients HCV PCR positive; (D) percent age distribution of patients anti-leptospire MAT positive; (E) percent age distribution of patients dengue positive (IgM, IgG and/or NS1).

Anti-HAV IgM was positive in 136 (35.8%) patients but the age distribution (Figure 1) indicated a surprisingly high proportion of older positive patients — 35% of those positive were ≥40 years old. Given the prior probability of high rates of exposure and childhood infection, IgG anti-HAV avidity assays were performed on sera from 51 anti-HAV IgM positive patients; 42 (82%) patients had IgG anti-HAV avidity >70%, suggesting polyclonal activation and not acute HAV.^6^, ^7^ Of the 10 patients with low IgG avidity 8 were HAV PCR positive, whilst none of those with avidity index >70% (42 patients) were HAV PCR positive. The HAV in all eight PCR positive patients was genotype 1A.

Using conservative definitions of disease and excluding HAV, 130/392 (33.2%) of patients had an aetiological diagnosis. Serology gave evidence for murine typhus in 14/382 (3.7%), scrub typhus in 8/382 (2.1%) and spotted fever group in 6/382 (1.6%) patients. Those with community-acquired septicaemia grew *S*. Typhi in five (1.6%), *E. coli* in five (1.6%), *Staphylococcus aureus* in two (0.6%) and *Burkholderia pseudomallei* in one (0.3%). Blood smears from the 68 patients originating from malaria endemic areas were all negative for *Plasmodium* spp. Four patients had evidence of previous infection with *C. burnetti* but no evidence was found for acute or chronic Q fever infection. No evidence for acute *N. sennetsu* was found.

Using the conservative criteria for diagnosis of mixed infections, 44 (11.2%) patients had evidence of mixed infections but the majority (61%) were HAV IgM positive (see Discussion). Of 136 patients with anti-HAV IgM, 20% had other diagnoses (Table 2). The remaining apparent mixed infections were mostly between leptospirosis/typhus and dengue/typhus.

**Table 2:** Evidence for dual positivity among serology, antigen and gene detection markers of acute jaundice/hepatitis among 392 Lao patients at Mahosot Hospital, 2001–2004. SFG = spotted fever group.

	HAV	HBV	HCV	HEV	Dengue	Leptospirosis	Scrub typhus	Murine typhus	SFG	Sepsis
HAV	**136**	6	1	1	0	9	2	3	2	3
HBV	6	**19**	0	2	0	0	0	0	0	0
HCV	1	0	**19**	0	0	0	0	0	1	2
HEV	0	0	0	**6**	0	1	0	0	0	0
Dengue	0	0	0	0	**33**	0	2	1	0	1
Leptospirosis	9	0	0	1	0	**26**	2	0	2	1
Scrub typhus	2	0	0	0	2	2	**8**	0	0	0
Murine typhus	3	0	0	0	1	0	0	**14**	0	0
Spotted fever group	2	0	1	0	0	2	0	0	**6**	0
Sepsis	3	0	2	0	1	1	0	0	0	**13**
All (% dual)	26/136 (19%)	6/19 (32%)	4/19 (21%)	4/6 (67%)	4/33 (12%)	15/26 (58%)	6/8 (75%)	4/14 (29%)	5/6 (83%)	7/13 (54%)

Past HBV infection was inferred in 61/388 (15.7%) patients by the presence of anti-HBsAg antibodies. Past HEV infection was inferred in 15.9% by the presence of anti-HEV total immunoglobulin without evidence of acute infection. Of the 119 patients with anti-HBsAg antibodies or HBsAg tested for HBc IgM, the peak age group with HBc IgM was the 25–29 year group (31%), declining to 9% in those over 60 years of age (Figure 1B). HCV antibody and PCR positive patients tended to be older: 74% of HCV PCR positive patients were >40 years old (Figure 1C). The frequency of patients with leptospirosis (Figure 1D) and rickettsia differed little across age groups but those positive for dengue infection were mostly younger: 67% were aged <30 years (Figure 1E).

The seroprevalence among 399 blood bank controls [mean (95% CI) age 22.3 (21.6–23.0) years; 80% male] of anti-HBsAg and anti-HCV antibodies were 6.5 and 0.25%, respectively. Of a subset of 198 controls [mean (95% CI) age 24.3 (23.3–25.4) years] anti-HEV antibodies were detected in 18.2%. Of a subset of 162 controls [mean (95% CI) age 20.2 (19.2–21.2) years] IgM anti-HAV was detected in none.

Two patients had serological evidence for acute HEV infection with HEV PCR confirmation. The first patient was a 27 year-old non-pregnant housewife from Xaysetta District, Vientiane City, with 6 days of fever and jaundice. She had left upper quadrant tenderness, total serum bilirubin 14.3 μmol/l, AST 55 IU/l and ALT 41 IU/l. The second patient was a 32 year-old construction worker from Sikkotabong District, Vientiane City, with four days of fever, jaundice, and right upper quadrant pain, a history of excessive alcohol intake, total serum bilirubin 530 μmol/l, AST 568 IU/l and ALT 321 IU/l. He was febrile, with spider naevi and hepatomegaly. Both were discharged well with supportive therapy. An additional four patients had significant antibodies against HEV but were HEV PCR negative.

The 26 leptospirosis serovars as determined by MAT were Pomona (1), Autumnalis (5), Bataviae (4), Javanica (3), Copenhageni (6), Grippotypphosa (3), Celledoni (1), Hardjo (1) and Mwalok (2). Some patients showed low level reactions against multiple serovars but determination of the infecting serovars could not be resolved.

### 3.2 Clinical features

Of all 392 patients the median (range) age was 34 (0.4–83) years, 259 (66%) were male and 381 (97%) were 15 years or older (Supplementary Table 1). Five patients with scrub typhus (Kato; admission IgG 1:512, IgM 1:1024 to IgG 1:1024, IgM 1:512, 5 days later), primary dengue (anti-dengue IgM positive), hepatitis B (admission anti-HBV core IgM), hepatitis A (admission anti-HAV IgM) and leptospirosis (MAT 1/1600 against Autumnalis) were pregnant. One patient had no aetiological diagnosis and none had acute HEV infection. All survived to discharge with intact pregnancies but the patient with HBV was discharged moribund.

Of 389 (99%) patients with discharge information 13 (3.3%) died in hospital; six (46%) had aetiological diagnoses of HCV infection (2), HAV infection (1), HBV infection (1), leptospirosis (1) and scrub typhus (1).

**Figure 2:**
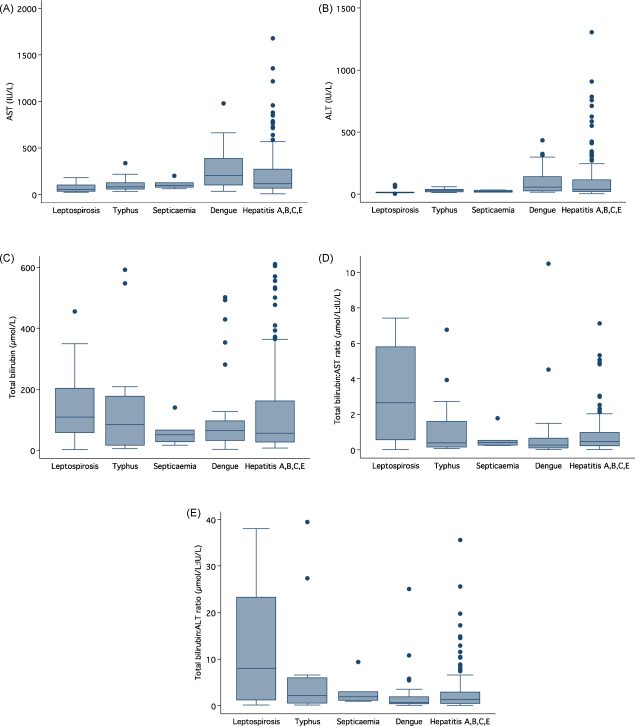
Box plots (median, 25th and 75th percentiles and whiskers to range) for serum AST (A), ALT (B), total bilirubin (C) concentrations and total bilirubin:AST (D) and total bilirubin:ALT (E) ratios for patients with laboratory evidence for leptospirosis, typhus (scrub typhus, murine typhus and spotted fever group combined), community-acquired septicaemia, dengue and viral hepatitis A,B,C and E. Patients with mixed infections have been excluded.

All viral A, B, C and E hepatitis patients’ data were combined and compared with those with leptospirosis, typhus, community-acquired septicaemia and dengue. Grossly raised transaminases was more common in the dengue and hepatitis A, B, C and E patients whilst high bilirubin was common for all disease categories except septicaemia (Figure 2 A-E). The total birirubin:AST ratio and total bilirubin:ALT ratios were higher for patients with leptospirosis and typhus than those with septicaemia, dengue and hepatitis A, B, C and E (Figure 2D and 2E). The clinical features of the 50 (12.8%, including mixed infections) patients with an infection expected to respond to doxycycline [leptospirosis and typhus-‘doxycycline responsive illness’ (DRI)] were similar to those with other causes of jaundice and raised transaminases, expect that those with DRI were more likely to have meningism (5/49, 10.2%) than those without (6/332, 1.8%) (*P* = 0.007). The median (range) AST was lower in those with DRI [88 (25–337) IU/l] than in those without [113 (2–1679) IU/l] and median (range) ALT was lower in those with DRI [23 (4–164) IU/l] than in those without [31 (2–1305) IU/l] (*P* = 0.008 and *P* = 0.003, respectively). The median (range) total bilirubin:AST and bilirubin:ALT ratios were significantly higher in those with DRI [0.818 (0.018–9.10) and 2.16 (0.116–39.48)] than in those without [0.507 (0.027–26.41) and 1.651 (0.067–66.58)] (*P* = 0.008 and *P* = 0.007, respectively). Applying multiple logistic regression to the 373 patients with data on meningism and serum AST and ALT, the odds ratios (95% CI) for DRI (against all without known DRI) were 5.3 (1.5–18.4, *P* = 0.009), 0.99 (0.99–1.00, *P* = 0.15) and 0.99 (0.98–1.00, *P* = 0.223), respectively. Inclusion of the bilirubin:AST/ALT ratios did not improve the models (*P* = 0.9 and *P* = 0.2, respectively). Considering those with and without leptospirosis, the median (range) AST was lower in those with this disease [85 (25–309) IU/l] than in those without [108 (2–1679) IU/l] (*P* = 0.01), median (range) ALT was lower in those with [15 (4–164) IU/l] than in those without [31 (2–1305) IU/l] (*P* = 0.003), the bilirubin:AST ratio was higher in those with [1.163 (0.018-9.095)] than in those without [0.513 (0.027–26.41] (*P* = 0.004) and the bilirubin:ALT ratio was higher in those with [3.433 (0.171–38.048)] than in those without leptospirosis [1.659 (0.067–66.58)] (P = 0.002).

None of the potential risks factors for jaundice/hepatitis (source of water, night soil use, past blood transfusions, intravenous drug misuse, sexually transmitted diseases, presence of rats, cats or pigs at home) were associated with any of the diseases tested for, except that those with rickettsial disease were less likely to have drunk from piped tap water (6/28) than those with other diseases (56/120) (*P* = 0.01).

## 4 Discussion

This study investigated the importance of nine viral and bacterial diseases/syndromes in the aetiology of patients presenting with jaundice and/or raised transaminases in Laos. As expected HAV, HBV, HCV and leptospirosis were identified as important causes. HBV immunization at birth and after has been introduced in Laos. Dengue was also common, emphasising the importance of considering dengue and appropriate specific fluid management.^14^ That 7.8% of patients who had blood cultures drawn had community-acquired bacteraemia, emphasises the importance of taking blood cultures in patients with biochemical liver derangement. In a series of patients at Mahosot Hospital with murine and scrub typhus, 34.6% had abnormal liver biochemistry^4^ and among 462 Japanese patients with scrub typhus elevated serum AST and ALT were also common (87% and 77%, respectively).^15^ The association of not drinking tap water with rickettsial disease presumably reflects that poorer people, without taps, are at greater risk.

The limitations of the study include that, as it was hospital based, it will tend to underestimate the incidence of disease in the community, that the median interval between acute and convalescent sera was relatively short at 6 days, that 48% of patients did not have a convalescent sample and that we did not investigate for HGV (GBV-C), parvovirus B19, syphilis, hantavirus, autoimmune diseases, medicine adverse effects and alcohol, and few children were included.^1^, ^16^ This study differs from that of Bounlu et al.^1^ which was a case–control study and included patients with <24 days of jaundice and dark urine, from three hospitals in Vientiane (including Mahosot Hospital). Falciparum malaria is likely to be more important as a cause of jaundice than suggested here in southern Laos where malaria remains common. We included patients with both jaundice and raised transaminases as reflecting abnormal biochemical liver function. However, raised bilirubin and transaminases can reflect pathology in other organs, potentially confounding the results.

We used conservative definitions of disease, which are appropriate for rickettsial disease, leptospirosis, septicaemia, dengue and HEV. However, we may have missed patients with acute HBV during the core window as we only performed HBcAb assays on patients who were HBsAg or anti-HBsAg positive. The only method to diagnose acute HCV infection conclusively is to document seroconversion and some of our HCV PCR positive patients may have had chronic, and not acute, HCV infection.^17^

An important finding is the description of diagnostic problems for HAV for the first time in the tropics.^6^, ^7^ Anti-HAV IgM, conventionally considered the gold standard for the diagnosis of acute HAV, persists for 3 months–5 years.^18^ However, HAV IgM can result from nonspecific polyclonal activation of memory cells and positive results not reflecting acute HAV infection may be more common than previously appreciated.^6^, ^7^, ^19^ The HAV IgG avidity and PCR analysis suggest that the majority of positive anti-HAV IgM results did not represent acute HAV infection but polyclonal activation. This is supported by the relatively low median transaminases in those with apparent HAV (Supplementary Table 1, Figure 2A and 2B) and the absence of anti-HAV IgM in blood donors. Hence, we are unable to provide a secure estimate of the incidence of acute HAV in this cohort. The diagnosis of HAV using IgM antibodies needs review and investigation of the causes of polyclonal activation. The predominant subgenotype in adjacent Thailand is also 1A.^20^

HEV has been described from adjacent countries^21^ and the six patients described here are the first confirmed from Laos. HEV infections are common in Lao domestic pigs^22^ and, if human HEV infections are contracted from pigs, HEV is likely to be a more important disease in rural Laos.

In adjacent northeast Thailand, HCV antibodies were detected in 6.5% of male (0.9% of female) blood donors and the prevalence of HBsAg was 4.9%. Of HCV antibody positive blood donors 80.4% were viraemic.^23^ In adjacent northern Vietnam, of 188 patients with clinical hepatitis, there was serological evidence for HAV in 29%, HBV in 24%, HCV in 10% with no evidence for acute HEV.^24^ Of HCV from 18 Lao patients in this study all were genotype 6, which has also been recorded in east and Southeast Asia.^11^ Although we found no evidence for acute *C. burnetii* infection, this has been recently described in northeast Thailand.^25^ In 1999–2003 a large epidemic of leptospirosis afflicted northeast Thailand, caused by Serotype 34 *Leptospira interrogans* serovar Autumnalis.^26^ However, only 5/26 (19%) of leptospira isolates from patients in Vientiane, 70 km from the study site of Thaipadungpanit,^26^ were Autumnalis. No epidemics of undifferentiated fever, such as occurred in adjacent areas of northeast Thailand, appear to have occurred in/around Vientiane. The Mekong River, which forms the Laos/Thai border in central Laos with only one bridge at the time of the study, may therefore have acted as an effective barrier preventing the significant spread of rats and the epidemic *Leptospira* clone into Laos.

The interpretation of apparent mixed infections, especially when based on serology for clinically similar diseases, is difficult. Given the difficulties demonstrated in the diagnosis of acute HAV, the apparent mixed infections with HAV may represent false HAV positivity. Those between leptospirosis and typhus and between dengue and typhus may represent serial infections with persistence of antibody, false positivity or genuine mixed infections. More research is required, using culture and antigen and gene detection tests, to tease out the relative frequency of these explanations in the Asiatic tropics.

Of the diagnoses entertained here, only leptospirosis, typhus and septicaemia, or 62 (15.8%, including mixed infections) patients, have specific affordable treatments available. In Thailand the current cost of treating HBV and HCV infections are approximately US$1000 and US$1250 a month, respectively. With a Laos per capita income of US$701 a year these are not affordable.^27^ Therefore, in the absence of locally accessible diagnostic tests and antiviral therapy in the vast majority of Laos, empirical therapy with doxycycline may be a cost-effective treatment with significant public health benefits for the 12.8% of patients with a DRI; these are currently the only patients with a potentially treatable disease. Those with a DRI represent 39% of all patients with a diagnosis and, excluding those with septicaemia, for the remaining 52% patients with a diagnosis, therapy is unavailable or unaffordable. A DRI is suggested by meningism and relatively low ALT (none of the patients with DRI had an ALT >164 IU/l). However, as transaminases assays are rarely available in Laos and relatively expensive (US$3.5 a test), empirical therapy for febrile jaundice with doxycycline may be cost effective, as the cost of a 7 day oral course of doxycycline is only approximately US$0.9 and is available in rural Laos. The available data suggest that doxycycline is safe in liver impairment.^28^ There is evidence that oral doxycycline, provided patients can swallow, is effective in severe leptospirosis and probably in severe typhus.^29^, ^30^ However, doxycycline adverse effects, especially to the upper gastrointestinal tract, are frequent and would reduce the positive public health impact of such an intervention.

## Authors’ contributions

BS, BR, VS, SP, RP, SS, TT, MM, NJW and PNN designed the study; BS, BR, VS, SP, SS, TT, MM recruited patients to the study; LS, JMR, AMR-A, KJ, VS, RP, TT, SDB, EB, PP, ED, DR, IH, PK performed the laboratory assays; PNN analysed the data; PNN, JMR, MM, EB, NJW drafted the paper and all authors revised, read and approved the final manuscript. PNN is guarantor of the paper.

## Funding

This study was funded by the Wellcome Trust of Great Britain. The work at the Peter Medawar Building for Pathogen Research was funded by the James Martin School for the 21st Century and the NIHR Biomedical Research Centre Programme.

## Conflicts of interest

None declared.

## Ethical approval

Ethical clearance was granted by the Ethical Review Committee of the Faculty of Medical Sciences, National University of Laos, Vientiane, Lao PDR.
