# Supplementary material for: The infective causes of hepatitis and jaundice amongst hospitalised patients in Vientiane, Laos
Source: Trans R Soc Trop Med Hyg. 2010 Jul;104(7-2):475–83. doi: 10.1016/j.trstmh.2010.03.002 (PMC2896487; doi:10.1016/j.trstmh.2010.03.002)
Supplement: Supplementary file 1 [file mmc1.doc]

**Supplementary Table 1**

**Clinical features of 392 Lao patients with jaundice and/or raised transaminases on admission**

| Variable | All | Leptospirosis | Murine typhus | Scrub typhus | Spotted fever group | Septicaemia | Dengue | HAV | HBV | HCV | HEV |
| --- | --- | --- | --- | --- | --- | --- | --- | --- | --- | --- | --- |
| No. | 392 | 26 | 14 | 8 | 6 | 13 | 33 | 136 | 19 | 19 | 6 |
| Age (years)^a^ | 34  (0.4–83) | 34  (17–65) | 35  (14–60) | 39  (20–60) | 51  (5–70) | 35  (16–70) | 23  (5–68) | 32  (8–83) | 27  (17–55) | 53  (5–72) | 30  (19–43) |
| Male (%) | 66 | 73 | 57 | 50 | 67 | 77 | 58 | 71 | 84 | 74 | 83 |
| % farmers | 8.7 ^391^ | 11.5 | 7.1 | 12.5 | 0 | 15.4 | 6.0 | 7.4 | 11.1 | 0 | 0 |
| No. days ill^a,b^ | 5 (1–28) | 3 (1–7) | 7 (2–15) | 10 (7–21) | – | 10 (3–20) | 6 (1–21) | 4 (1–21) | 2 (1–14) | 7 (3–11) | 3 (1–5) |
| Fever (%) | 69.6 ^391^ | 77.0 | 71.4 | 75.0 | 83.3 | 92.3 | 84.8 | 77.2 | 52.6 | 68.4 | 83.3 |
| Headache (%) | 46.8 ^391^ | 50.0 | 42.9 | 50.0 | 50.0 | 46.2 | 54.5 | 51.9 ^135^ | 36.8 | 42.1 | 50.0 |
| Jaundice (%) | 86.9 ^390^ | 92.3 | 71.4 | 87.5 | 83.3 | 84.6 | 81.8 | 90.4 ^135^ | 94.7 | 88.8 ^18^ | 100 |
| Vomiting (%) | 26.9 ^391^ | 38.5 | 28.6 | 25.0 | 16.7 | 23.1 | 39.4 | 35.6 ^135^ | 0 | 5.3 | 0 |
| Diarrhoea (%) | 21.2 | 30.8 | 7.1 | 25.0 | 33.3 | 46.2 | 18.2 | 23.5 | 11.8 | 26.3 | 0 |
| Abdominal pain (%) | 57.1 | 61.5 | 64.3 | 50.0 | 50.0 | 69.2 | 39.4 | 64.7 | 57.9 | 42.1 | 50.0 |
| Temperature (°C) ^c^ | 37.5 ^324^  (37.4–37.6) | 37.2  (36.8–37.5) | 37.5 ^11^  (36.7–38.3) | 37.6  (36.2–38.9) | 37.7  (36.5–38.8) | 37.8 ^10^  (37.0–38.5) | 37.7  (37.3–38.2) | 37.5 ^112^  (37.4–37.7) | 37.1 ^18^  (36.6–37.5) | 37.3 ^18^  (36.8–37.8) | 37.5  (36.5–38.5) |
| Liver palpable (%) | 57.5 ^381^ | 56 ^25^ | 46.2 ^13^ | 62.5 | 40.0 | 41.7 ^12^ | 55 ^31^ | 62.6 ^131^ | 52.6 | 36.8 | 33.3 |
| Spleen palpable (%) | 10.5 ^381^ | 28 ^25^ | 15.4 ^13^ | 12.5 | 20.0 | 0 | 19 ^31^ | 8.4 ^131^ | 0 | 0 | 0 |
| Confused (%) | 4.9 ^385^ | 8 ^25^ | 7.1 | 25.0 | 0 | 0 | 0 | 4.5 ^132^ | 5.3 | 0 | 0 |
| Rash (%) | 3.1 ^385^ | 0 | 7.1 | 0 | 0 | 0 | 3 ^32^ | 2.3 ^129^ | 0 | 0 | 0 |
| Urobilinogen in urine (%) | 33.3 ^381^ | 28 ^25^ | 42.9 | 50.0 | 33.3 | 30.8 | 45.5 | 34.8 ^135^ | 44.4 ^18^ | 31.6 | 50.0 |
| Bilirubin in urine (%) | 51.8 ^382^ | 48 ^25^ | 57.1 | 75.0 | 33.3 | 23.1 | 39.4 | 57.0 ^135^ | 88.8 ^18^ | 52.6 | 100.0 |
| Creatinine  (μmol/l)^a,d^ | 99 ^375^  (27–1452) | 134 ^24^  (62–1046) | 81  (35–134) | 115  (74–425) | 101  (72–125) | 114  (62–263) | 95 ^32^  (35–453) | 102 ^129^  (27–1452) | 76  (49–162) | 106  (54–248) | 79  (50–106) |
| Urea (mmol/l)^a,e^ | 4.7 ^375^  (1.8–71.1) | 8.4 ^24^  (2.1–48.8) | 3.9  (1.8–7.7) | 8.1  (2.3–37) | 4.6  (3.5–9.7) | 5.3  (2.7–31.7) | 4.1 ^32^  (1.9–18.5) | 4.4 ^129^  (1.9–71.1) | 4.8  (2.2–11.3) | 4.9  (2.0–16.0) | 2.8  (2.3–20.3) |
| Total bilirubin (μmol/l)^a,f^ | 66 ^385^  (2–999) | 82  (2–503) | 89  (51–548) | 139  (32–592) | 42  (17–73) | 57  (16–479) | 67  (3–502) | 56 ^133^  (7–570) | 234  (22–611) | 52  (14–340) | 379  (14–604) |
| Direct bilirubin (μmol/l)^a,g^ | 34 ^385^  (2–559) | 54  (2–280) | 47  (2–269) | 97  (17.6–300) | 20  (7–57) | 24  (6–357) | 36  (2–291) | 30 ^133^  (2–357) | 129  (15–405) | 27  (5–188) | 249  (7–332) |
| AST (IU/l)^a,h^ | 106 ^385^  (2–1679) | 85  (25–309) | 81  (30–248) | 146  (37–337) | 98  (69–213) | 87  (60–376) | 199  (33–977) | 104 ^133^  (7–1679) | 313 ^18^  (115–848) | 155  (40–879) | 252  (55–568) |
| ALT (IU/l)^a,i^ | 30 ^385^  (2–1305) | 15  (4–164) | 37  (14–145) | 27  (15–164) | 29  (15–75) | 31  (14–245) | 55  (15–432) | 32 ^133^  (2–1305) | 82  (15–907) | 30  (15–143) | 62  (15–321) |
| AST and/or ALT threefold increase (%) | 52 ^385^ | 27 | 35.7 | 62.5 | 33.3 | 38.5 | 76 | 49.6 ^133^ | 100 | 68.4 | 100 ^5^ |
| Alkaline phosphate (IU/l)^a,j^ | 149 ^385^  (14–1610) | 110 ^24^  (45–918) | 159  (54–667) | 216  (70–918) | 190  (59–479) | 222  (115–1610) | 108 ^31^  (14–580) | 147 ^126^  (35–908) | 163  (72–550) | 131 ^18^  (66–515) | 140  (80–170) |
| Albumin (g/l)^a,k^ | 37 ^374^  (13–59) | 34 ^24^  (22–48) | 46  (24–57) | 29  (17–44) | 32 (23–39) | 33  (25–48) | 41 ^32^  (17–54) | 39 ^129^  (13–57) | 37  (19–49) | 30  (14–44) | 39  (20–58) |
| Hematocrit (%)^a,l^ | 35 ^244^  (7–60) | 35 ^17^  (14–50) | 31 ^6^  (22–40) | 35 ^3^  (24–39) | 37 ^4^  (32–42) | 32  (21–48) | 38 ^21^  (11–56) | 38 ^86^  (19–60) | 38 ^14^  (28–53) | 28 ^13^  (18–45) | 39 ^3^  (35–40) |
| White cell count (10^9^/l)^a,m^ | 10.6 ^244^  (3.2–38.5) | 12.6 ^17^  (5.2–20.7) | 11.8 ^6^  (6.6–22.6) | 13.8 ^3^  (9.8 –15.8) | 11.3 ^4^  (5.2–17) | 11.4  (3.5–32) | 10.7 ^21^  (3.4–25) | 10.6 ^86^  (3.2–34.8) | 9.4 ^14^  (3.2–22.6) | 7.5 ^13^  (4.3–22.7) | 8.8 ^3^  (6.2–13.3) |
| Platelets (10^9^/l)^a,n^ | 190 ^220^  (6–742) | 180 ^15^  (6–250) | 184 ^6^  (170–210) | 200 ^3^  (190–210) | 180 ^4^  (45–250) | 195 ^12^  (70–571) | 180 ^21^  (30–320) | 200 ^77^  (55–566) | 200 ^14^  (51–294) | 180 ^13^  (45–426) | 120 ^3^  (20–180) |

^a^ Median (range).

^b^ Excluding those recorded as ill for >30 days.

^c^ Mean (95% CI).

Reference ranges: ^d^ 62–120 μmol/l, ^e^ 5.4–16.0 mmol/l, ^f^ 3–14.5 mol/l, ^g^ 0–4.3 mmol/l, ^h^ <37 IU/l, ^i^ <40 IU/l, ^j^ 120–290 IU/l, ^k^ 38–54 g/dl, ^l^ males 40–48% and females 37–42%, ^m^ 4.0–11.0 10^9^/l, ^n^ 150–400 10^9^/l. Superscripts for variables with missing values give the available sample size for that variable.
